# Supplementary material for: Chromothripsis during telomere crisis is independent of NHEJ, and consistent with a replicative origin
Source: Genome Res. 2019 May;29(5):737–49. doi: 10.1101/gr.240705.118 (PMC6499312; doi:10.1101/gr.240705.118)
Supplement: Supplemental Material [file supp_gr.240705.118_Supplemental_file_1.zip › contigs/annotated_contigs/DB38/contig.2.DB38_length_634_mean_cov_7.1214511041.docx]

**DB38_length_634_mean_cov_7.1214511041**

```
CCGGCCCGTGGGTGTGGCATTGAAGAAAGTCACTGGTGCCTGGGAGAAGGACGCTGATTGGGCCAGGGCAGAGTCTGGCAGAAGAGGGG
 >chr6:43413034-43413375 - E=2e-193
TTGGCAGGCAGCACTGGGGGAGGTGGGGTGGAGGGGAAGGCCCAAGATAGAGAATAGGGAAGGACTAAAATATCCCCAGAATTTGGGGG

CAGAGAAGAGAGATTGGCAGAGGTTGAGGAAGAATATCCCAGGGCAGGAAAGACTCTCCCATCCCCAGTGGAGCCCGAGTATTGGGAGG

ACACTCCACAGGGGCTAGGACTGCACTCCCCTACCCCCGAGGTCCCAGCCCCTCACCATAAGGACTCGATGCA|G|TAGTCCCAGCTAC
                                                                          >chr6:4340754
TCAGGAGGCTGAGGCAAGAGAATTACTTGAACCCGAGCGGCAAAGGTTGCAGTAAGCCGAGATCATTCCACTGCACTCCAGCCTGAGCG
1-43407835 - E=3e-165
ACAGAGCAAGACTCTGTCTCACACACACGAAAAAGTAAAAACCTATAAAAGCTTTTACATAAAATCTGCCCAAGATATCTTACTACCTT

GACTGTTAAAAAGACAGGGTAGTAGGCCAGGCGCGGTGGCTCACGCCTGTAATCCCAGCACCTTGGGAGGCCAAGGTGGGCAGATCACA

AGGTCAGGAGATG
```
